# Pregnancy and contraceptive questioning within acute inpatient psychiatric admissions: are we asking enough?

**DOI:** 10.1192/bjo.2021.171

**Published:** 2021-06-18

**Authors:** Eleanor Partington

**Affiliations:** Newcastle University, Cumbria, Northumberland, Tyne And Wear NHS Foundation Trust

## Abstract

**Aims:**

This audit explored how regularly women of childbearing age on an acute psychiatric inpatient ward are asked about pregnancy and contraception.

**Background:**

Unplanned pregnancies and poor compliance with contraception are common in women with severe mental illness, with a significant number seeking abortions or losing custody of their children. As these women are also less likely to consult medical professionals, an admission is an essential point for intervention and support.

Additionally, there are risks associated with prescribing psychotropic medications during pregnancy. Because of this, The Royal College of Psychiatrists and local guidelines state that all female patients admitted onto psychiatric inpatient wards should be asked about their sexual health within seven days of admission.

**Method:**

Data was collected from all 51 women of childbearing age admitted to a mixed-sex, acute adult inpatient psychiatry ward over one year, from January 2019 until the end of December 2019.

Women of childbearing age were deemed to be those between the ages of 15 and 45, based on the World Health Organization's definition. However, the sample for this audit includes females aged 18–45 years due to the minimum age restrictions of the ward.

All eligible female inpatients had their physical health forms and progress notes screened for documentation of whether a) the possibility of them being pregnant was explored b) if a pregnancy test was done and c) if a contraceptive history was taken.

**Result:**

Only 57% of female patients admitted during this period were asked about their contraceptive habits. Furthermore, exploration into the possibility of pregnancy occurred in less than half of admitted patients.

Further analysis was done by age; 18-26, 27-35 and 36-45, but showed minimal variation.

**Conclusion:**

This audit revealed that Royal College of Psychiatrists and local guidelines are not being met, with women not receiving the recommended assessment and counselling in regard to pregnancy and contraception.

Inpatient admissions provide a valuable opportunity for identifying and preventing potential harm in the case of unplanned and undetected pregnancies. All health care professionals need to be aware of the importance of asking the above questions and ensure they are explored at some point during a patient's admission.

The audit will be discussed at forthcoming Clinical Governance meeting for further recommendations followed by re-audit.

